# Causes and Evolutionary Consequences of Population Subdivision of an Iberian Mountain Lizard, *Iberolacerta monticola*

**DOI:** 10.1371/journal.pone.0066034

**Published:** 2013-06-07

**Authors:** Nuria Remón, Pedro Galán, Marta Vila, Oscar Arribas, Horacio Naveira

**Affiliations:** 1 Departamento de Bioloxía Animal, Bioloxía Vexetal e Ecoloxía, Facultade de Ciencias, Universidade da Coruña, A Coruña, Spain; 2 Departamento de Bioloxía Celular e Molecular, Facultade de Ciencias, Universidade da Coruña, A Coruña, Spain; 3 Avda. Francisco Cambó 23, Barcelona, Spain; Tuscia University, Italy

## Abstract

**Aim:**

The study of the factors that influence population connectivity and spatial distribution of genetic variation is crucial for understanding speciation and for predicting the effects of landscape modification and habitat fragmentation, which are considered severe threats to global biodiversity. This dual perspective is obtained from analyses of subalpine mountain species, whose present distribution may have been shaped both by cyclical climate changes over ice ages and anthropogenic perturbations of their habitats. Here, we examine the phylogeography, population structure and genetic diversity of the lacertid lizard *Iberolacerta monticola*, an endemism considered to be facing a high risk of extinction in several populations.

**Location:**

Northwestern quadrant of the Iberian Peninsula.

**Methods:**

We analyzed the mtDNA variation at the control region (454 bp) and the cytochrome *b* (598 bp) loci, as well as at 10 nuclear microsatellite loci from 17 populations throughout the distribution range of the species.

**Results:**

According to nuclear markers, most sampling sites are defined as distinct, genetically differentiated populations, and many of them show traces of recent bottlenecks. Mitochondrial data identify a relatively old, geographically restricted lineage, and four to six younger geographically vicariant sister clades, whose origin may be traced back to the mid-Pleistocene revolution, with several subclades possibly associated to the mid-Bruhnes transition. Geographic range fragmentation of one of these clades, which includes lowland sites, is very recent, and most likely due to the accelerated loss of Atlantic forests by human intervention.

**Main Conclusions:**

Altogether, the data fit a “refugia within refugia” model, some lack of pattern uniformity notwithstanding, and suggest that these mountains might be the cradles of new species of *Iberolacerta*. However, the changes operated during the Holocene severely compromise the long-term survival of those genetic lineages more exposed to the anthropogenic perturbations of their habitats.

## Introduction

The Quaternary period is punctuated by a series of cyclic large glacial-interglacial climate changes, particularly intense in the northern hemisphere, primarily determined by parameters of the Earth’s orbit [1]. Longer cold and predominantly dry periods in Europe alternate with others much shorter and warmer, whose effects on the evolution of species are strongly influenced by central and south high mountains (European Alpine system), and may vary considerably among different ecological and distributional groups [2]. In principle, the influence of middle high mountains in shaping the changes of a species’ range should be comparatively small, yet the phylogeographic patterns at these lower sites (below the upper level of tree growth, *i.e.* at subalpine and forest zones) may be markedly different from those of alpine species, and serve for a better understanding of the conditions that ultimately lead to speciation [3]. Vicariance episodes, so inextricably linked to these climate changes, bring up for consideration the effects of habitat fragmentation on the genetic properties of these populations, particularly with respect to their evolutionary potential and, with climate amelioration, their capacity to expand from refugia. Isolated populations resulting from fragmentation, especially if they become sufficiently small, must face both deterministic (edge and Allee effects) and stochastic threats (environmental and demographic) [4]–[6], which determine minimum threshold densities and may eventually drive fragmented populations into “extinction vortices” [7], or “mutational melt-downs” [8].

**Figure 1:**
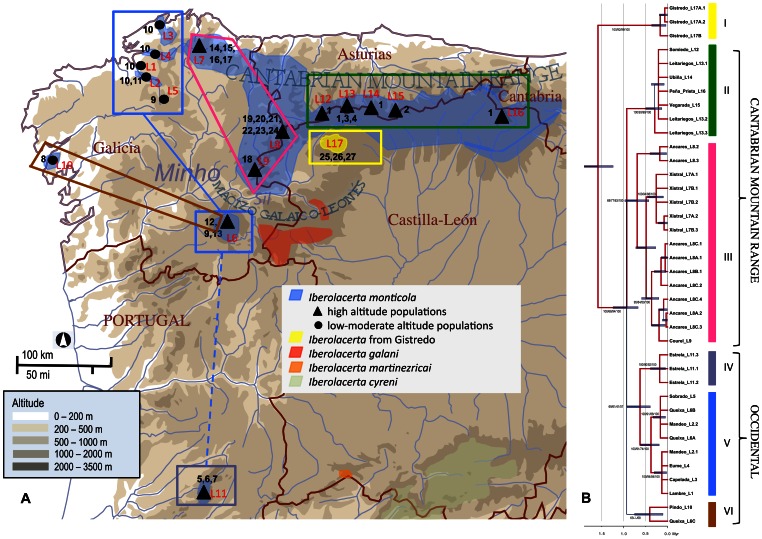
Mapping of the mitochondrial phylogeny of *I. monticola* on the geographical distribution of the species. Localization of the samples and mitochondrial haplotypes of *I. monticola* at the NW of the Iberian Peninsula. **B)** Mapping on this geographical distribution of the Bayesian phylogenetic tree of the mitochondrial sequences. A geological time scale in millions of years is shown below. Abbreviations: L1– Lambre; L2– Mandeo; L3– Serra da Capelada; L4– Eume; L5– Sobrado; L6– Serra da Queixa; L7– Serra do Xistral; L8– Serra dos Ancares; L9– Serra do Courel; L10– Montes do Pindo; L11– Serra da Estrela; L12– Somiedo; L13– Leitariegos; L14– Ubiña; L15– Vegarada; L16– Peña Prieta; L17– Sierra de Gistredo. Limits and corresponding nouns of the main administrative divisions of the study area are indicated on the map. Labels marking the extent and situation of the main river basin (Minho-Sil) and the two main mountain systems (Cantabrian Mountain Range and Macizo Galaico-Leonés) of this area are also placed on the map. Numbers from 1 to 23 denote mitochondrial haplotypes, as in Figure 2. The tree is rooted using *I. cyreni* (see Fig. S1). Range between min and max posterior probability values (0.53–1.0) is indicated by color and width gradients (blue to red, and slim to thick). Support values obtained by four different methods of analysis for the major clades of *I. monticola* (II, III, IV-V, and VI) are shown at each node; namely, from left to right, Bayes posterior probability (×100), ML best trees in consensus (%), NJ-bootstrap (%), and equally MP trees (%). A hyphen was inserted instead of a numerical value whenever a particular method did not support the Bayesian topology.

*Iberolacerta monticola* (Boulenger 1905) is one of the species included in the Iberian rock-lizard group, whose phylogenetic relationships and evolutionary histories are relatively well known [9]–[14]. It is defined as “vulnerable” [B1ab(iii)] in the IUCN Red List of Threatened Species, according to its extent of occurrence (less than 20,000 km^2^), its distribution (severely fragmented), and the quality and extent of its habitat (in continuing decline) [15]. Endemic to the NW quadrant of the Iberian Peninsula, it is nominally subdivided into *I. monticola monticola*, restricted to the Serra da Estrela in Portugal, within the Western Mediterranean region of the Peninsula, and *I. m. cantabrica*, distributed across a wide area in NW Spain, within the Atlantic biogeographic region, mainly at rocky habitats in subalpine and forest zones of the Cantabrian Mountain Range. The mean height of these mountains falls off considerably after the so-called Picos de Europa massif, and Sierras de Peña Prieta, Peña Sagra and Híjar, on entering Cantabria, which turns out to be a determinant feature of the eastern limit of the *I. m. cantabrica* distribution (Figure 1A). Western populations, however, can be found at downright lowland areas, most of them associated to patches of Atlantic forests in shady fluvial gorges of Galicia [16]. Besides, whereas the species extends with no apparent discontinuities over vast areas of suitable habitat throughout the Cantabrian Mountains, it appears severely fragmented elsewhere, with evidences of ongoing range contraction and local population extinction [17]–[19]. On the other hand, glacial dynamics in the NW of the Iberian Peninsula [20], [21] indicate that large parts of the current range of *I. monticola* at middle high altitude (in principle, most sites >700–1,000 m asl, meters above sea level) must have been unsuitable for the species during the last ice age, a situation that most likely took place repeatedly during the cyclical climate changes of the Pleistocene [22].

Molecular data openly question the subspecific rank of the population from Serra da Estrela and, conversely, point out an unexpectedly high degree of differentiation between the populations of Galicia and the Cantabrian Range, but fail to produce conclusive evidence on the location of putative glacial refugia, or the eventual dispersal routes from them [9]–[12], [14], [23]. The present study is thus aimed first at depicting and understanding the phylogeographic patterns of the populations of *I. monticola*, which may have been shaped both by Pleistocene glacial cycles and Holocene habitat fragmentation. But then we would like to address several issues concerned with the conservation of this species, by assessing the effects of population decline on the levels of deleterious mutation load and evolutionary potential to respond to an ever-changing environment.

## Methods

### Sampling, Microsatellite Genotyping and mtDNA Sequencing

Sampling of *I. monticola* was designed to include a full representation of the known distribution of this species. Ethics approval of all procedures involving vertebrate animals is legally required under the Spanish legislation (Royal Decree 1201/2005 and Law 32/2007, on the protection of animals used for experimentation and other scientific purposes), which is a transposition of the European Directive 86/609/EEC. In agreement with article 18 and annexes VII and XI of the said Royal Decree, all animal procedures performed as part of the experimental work described in this paper have received prior and explicit approval from the competent authorities, defined in article 3e of the Law, and substantiated in the corresponding regulations of the Spanish autonomous communities. Thus, permissions for fieldwork and the concomitant experimental procedures were issued by the *Xunta de Galicia*, *Junta de Castilla-León*, and *Principado de Asturias*, in Spain, and, in application of an analogous transposition of the European Directive, by the *Instituto da Conservaç*ã*o da Natureza* in Portugal. Altogether, 316 lizards were genotyped, from 17 populations and 24 sites covering most of the species range (Table S1 and Fig. 1A). In every case, animals were quickly (<5 min) processed at the capture site and immediately released afterwards. Processing consisted of obtaining biometric data and collecting tail-tips, which were preserved in 96% ethanol for molecular studies. Genetic variation was analyzed at 10 nuclear (microsatellite) and two mitochondrial loci (*cytb*–cytochrome *b*, 570 bp; CR–control region, 453 bp), corresponding to position intervals 14,193–14,763 and 16,311–16,773, respectively, of the *Lacerta viridis* mitochondrial genome (GenBank acc. no. AM176577); for amplification details, see Table S2. Whereas mitochondrial data were obtained from all the visited sites, the microsatellite analysis was restricted to *I. monticola* populations, and then to 15 representative localities (Table S1; data deposited at Dryad: http://dx.doi.org/s2479). This is made explicit in this section, since two of the sampling sites happened to harbor not *I. monticola*, but a different, so far undescribed, new *Iberolacerta* taxon (see Results). DNA extractions from tissue samples and genotyping were carried out as described elsewhere [24]. Both mitochondrial markers were bidirectionally sequenced for 5–10 specimens per locality, and electropherograms were visualized and aligned using CodonCode Aligner v. 3.5.7 (CodonCode Corporation). Newly reported sequences have been deposited in GenBank (acc. nos. HQ234877–HQ234900, and EF121827–EF121834; see also Table S1 for a full cross reference of haplotypes and GenBank accessions). Sequences from two other closely related *Iberolacerta* species, namely *I. galani* and *I. martinezricai*, were included in the analyses (GenBank acc. nos. EF121835, EF121836, HQ234882, HQ234901, HQ234902 and HQ234903). A sequence from *I. cyreni* (constructed from EF121834 and AY267232) was used as outgroup to root the phylogenies.

### Microsatellite Data Analyses

The MicrosatelliteToolKit [25] was used to format data input for other software used in this work, as well as to obtain frequencies of heterozygotes, both observed (*h*_obs_) and expected under Hardy-Weinberg equilibrium (*h*_HW_), and numbers of alleles for each locus and population (*n_a_*), together with the corresponding averages across loci for each population (*H*_obs_, *H*_HW_, and 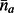). Exact tests of Hardy-Weinberg equilibrium (HWE) for each sample were conducted with Genepop v. 4.0 [26]. Genetic structure within populations was examined by the inbreeding coefficient (*f_IS_*, or *F*_IS_ when averaged over all loci) [27]. Randomization based tests carried out with the aid of Fstat v. 2.9.3 [28] were used both to assess the significance of *f_IS_* (1500 randomizations, for each locus in each population), and possible non-random allelic associations between pairs of polymorphic loci (2100 permutations). The significance level of all these tests was adjusted by the sequential Bonferroni procedure [29]. Fisher’s exact tests of genic differentiation for all pairs of populations were conducted with Genepop v. 4.0.

We checked the analyzed populations for evidence of recent bottlenecks using both Bottleneck v. 1.2.02 [30] and *M*-ratio software [31]. In the approach implemented in Bottleneck, a two-phase mutation model with 5% of multi-step changes was assumed, using 10,000 iterations, and Wilcoxon signed rank tests of significance. For the *M*-ratio test, the bottleneck hypothesis was tested under three different population scenarios, corresponding to different values of the mutation parameter *θ* (4*Ne*µ), as in [32], namely *θ = *value estimated from the data, *θ = *10, and *θ = *0.5. See footnote at Table 1 for definitions, abbreviations and further methodological details.

**Table 1:** Signatures of population bottlenecks on microsatellite variation.

				Bottleneck			*M*-ratio			
Label	Population	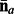	*F*_IS_	*H*_HW_	*H*_MD_	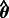	*m* (*P*-value)	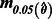	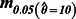	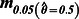
L1	Lambre	4.09	0.012	0.618	0.587	2.9	**0.654** (0.011)	0.770	0.691	0.859
L2	Mandeo	6.09	−0.001	0.686	0.717	4.6	0.749 (0.075)	0.771	0.738	0.864
L3	Capelada	5.27	0.095	0.737	0.721	6.7	0.686 (0.089)	0.702	0.670	0.854
L4	Eume	7.09	0.082	0.761	0.773	8.2	0.766 (0.216)	0.730	0.718	0.862
L5	Sobrado	4.00	0.052	0.547	0.546	1.9	**0.598** (0.000)	0.819	0.756	0.866
L6A	Queixa	3.60	0.039	0.595	0.585	2.7	**0.624** (0.008)	0.768	0.668	0.859
L7	Xistral	7.00	0.061	0.743	0.759	7.1	0.742 (0.087)	0.742	0.729	0.862
L8A	Ancares	9.18	0.072	0.847	0.841	20.8	0.834 (0.726)	0.660	0.707	0.861
L9A	Courel	6.80	0.104	0.744	0.738	6.6	0.729 (0.098)	0.734	0.707	0.861
L9B	Courel	6.50	0.101	0.752	0.761	7.9	**0.646** (0.005)	0.715	0.702	0.862
L10	Pindo	5.55	−0.012	0.637	0.576	3.3	**0.720** (0.009)	0.794	0.757	0.868
L11	Estrela	7.18	0.008	0.733	0.798	6.5	0.866 (0.878)	0.735	0.715	0.863
L13	Leitariegos	8.00	0.018	0.781	0.763	10.3	0.794 (0.198)	0.750	0.751	0.864
L15	Vegarada	5.00	0.000	0.655	0.672	3.7	**0.642** (0.005)	0.762	0.694	0.861

Intrapopulation genetic diversity indices (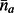, *F*_IS_, and *H*_HW_) and results of two tests for population bottlenecks (Bottleneck and *M*-ratio), based on averages across microsatellite loci.

Abbreviations: 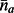 – observed mean number of alleles per locus; *F*_IS_ – observed mean inbreeding coefficient; *H*_HW_ – expected heterozygosity at Hardy-Weinberg equilibrium; *H*_MD_ – expected heterozygosity at mutation-drift equilibrium, assuming a two-phase model (TPM) of mutation; 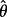
–estimate of the population mutational parameter; *m* – observed mean ratio of allelic size range against total number of alleles; *P*-value –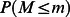, from an equilibrium distribution; *m*_0.05_ – critical *M* values 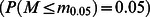 for equivalent samples obtained from populations at mutation-drift equilibrium under different scenarios of mutational input per generation (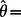 value estimated from the data, 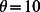, 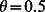).

*M*-ratio results that turned out to be significant under all three scenarios are shown in bold.

Two geographically “blind” approaches (*i.e.*, with no *a priori* assumptions about populations) were taken to visualize the similarities among multilocus genotypes. First, by constructing a UPGMA tree of individuals [33], based on pairwise allele-sharing genetic distances, and then by performing a factorial correspondence analysis (FCA). The matrix of allele-sharing distances [34] was obtained with the aid of the microsatellite analyser (MSA) v. 4.05 [35]. The corresponding tree was generated by the Neighbor program in PHYLIP v. 3.6 [36], and then exported to Mega 4 [37], just for editing and printing purposes. FCA was carried out with Genetix v. 4.05.2 [38].

At the population level, we first constructed a neighbor-joining tree of the populations, based on Cavalli-Sforza & Edwards chord distance, *D*_C_, which performs best for reconstructing tree topologies in simulated microsatellite data [39]. Support for tree nodes was obtained by bootstrapping loci 10,000 times. Bootstraps samples and chord distances were obtained with MSA. As a second approach at this level of organization, discrimination among populations was then inferred using the Bayesian assignment procedures implemented in the software Structure v. 2.1 [40]. To identify the likely number of populations (*k*) within *I. monticola*, we used both the method suggested in the original Structure paper, based on scoring mean log likelihoods penalized by one-half of their variance (estimated “log probability of data”, *L*(*k*)), and the approach developed by Evanno et al. [41], based on the rate of change in the log probability of data between successive *k* values (Δ*k*). We used an admixture model of genetic clustering with correlated allele frequencies, run for 50,000 generations after a burn-in of 100,000 generations, assuming that there were up to 14 possible partitions of the data (*k = *2 to *k = *16), and ran 10 parallel chains to estimate what number of genetic clusters had the highest probability. Membership coefficients of individuals to each of the clusters were plotted with Distruct v. 1.1 [42], with individuals ordered geographically, generally from the western to the eastern parts of the range.

### Mitochondrial DNA Data Analyses

The topological congruence between the phylogenies obtained with the two markers was assessed by the incongruence length difference (ILD) test [43], [44], carried out with the aid of PAUP* v. 4.0 [45]. Maximum parsimony (MP), neighbor-joining (NJ), maximum likelihood (ML) and Bayesian methods were used for reconstruction of mitochondrial phylogenetic trees. MP and NJ analyses were conducted in Mega 5 [46], which also provided several descriptive statistics (number of variable and parsimony informative sites, *p*-distances between sequences). MP trees were obtained using the Close-Neighbor-Interchange algorithm, with search level 3, and 100 random addition sequence replicates. The NJ tree was based on distances obtained by the Tamura-Nei model, allowing for a heterogeneous pattern among lineages and gamma-distributed rates among sites. The value of the gamma shape parameter (0.20) was obtained using a combination of ancestral sequence inference and maximum likelihood estimation [47], with the aid of GZ-Gamma (http://www.personal.psu.edu/nxm2/Software/gamma/gamma.zip), based on 10,000 bootstrap replicates. For ML, we first determined the model of sequence evolution that best fitted each of the four partitions of the data, namely (*i*) control region, (*ii*) 1st, (*iii*) 2nd, and (*iv*) 3rd codon positions of the *cytb*, by the Bayesian Information Criterion (BIC), using the metaPIGA v.2.1.3 development (http://www.metapiga.org) [48] of jModelTest [49]. ML phylogeny for the combined mitochondrial dataset was estimated by consensus pruning (meta-population genetic algorithm), using default operators and parameters in metaPIGA, choosing the best-fit model selected by BIC for each partition (HKY85+ Γ, with the corresponding estimates of the shape parameter). We used loose Neighbor-Joining (20% range) to generate the starting trees, based on a HKY85 distance matrix, with rate heterogeneity across sites (shape parameter of the Γ distribution = 0.20). A ML test of the molecular clock hypothesis for the consensus topology thus obtained was carried out with Mega 5. As for the Bayesian phylogenetic inference, we used MrBayes v. 3.2 [50], [51], again specifying a HKY85+ Γ model for each of the four partitions of the data. The analysis was carried out with MrBayes default priors until the standard deviation of split frequencies dropped below 0.01, and the potential scale reduction factor for all parameters lied close to 1.0. Two simultaneous, completely independent analyses starting from different random trees were run. For the Markov chain Monte Carlo (MCMC) sampling of the target distribution, three heated chains and one cold chain were used. The first 25% samples from the cold chain were discarded as burn-in. We used Bayes factor comparisons to test several topological hypotheses. Marginal model likelihoods were estimated by the stepping-stone method; strength of the evidence in favor of the better model was then assessed by the magnitude of the log-difference, following Kass and Raftery [52]. The strict clock model was tested against the non-clock model using an analysis similar to the previous one, by comparing the marginal likelihoods of the two models. For tree calibration and dating, we used a uniform prior from 6.5 to 8.5 My on the oldest split in the tree, corresponding to the separation of *cyreni*, and from 1.9 to 2.9 My on the *monticola-galani-martinezricai* split. These “hard” bounds are based on Carranza et al. (2004) [12] (see also [10], [53]), whose dating method, relying on a single calibration point, has been reported to underestimate the age of several genera of Lacertini, as well as the time for the separation of major lineages of Lacertidae [54]. However, the estimates for the divergence of other, relatively younger, genera of Lacertini (including *Iberolacerta*) fall well within the 95% confidence intervals produced by nearly all the methods and models tested by Hipsley et *al.* (2009) [54]. MCMC analysis was run using 1,000,000 generations. The output cladogram summarizing the trees was visualized with FigTree v. 1.3.1 [55]. Mean ages and 95% highest posterior density intervals of mtDNA phylogroups are used as estimates of divergence times.

Since tree-building methods tend to resolve intraspecific gene genealogies poorly when the different mitochondrial types are separated by few mutations, and ancestral haplotypes are still present in the populations [56], a network was also generated through tcs v. 1.21 [57] and its outcome compared to the previous phylogenies.

The Alignment Transformation EnviRonment (ALTER) [58] was used to obtain properly formatted input data for the different programs used in this part of the work.

### Mantel Tests and Analysis of Molecular Variance

Genetic samples were separated into two groups, corresponding to *a priori* continuous or fragmented distributions of the species (see Fig. 1A). Pairwise genetic differentiation between populations within each group was estimated both by *F*_ST_ [27] and *D*_C_ [59] statistics, based on microsatellite frequencies (obtained with MSA), and by φ_ST_ [60] for corresponding mtDNA data (obtained as part of the analysis of molecular variance, described below). To evaluate the relative importance of philopatry and population fragmentation in predicting levels of genetic structure across the study region, we then conducted a series of Mantel tests [61] with the aid of ibdws v. 3.15 [62], using 10,000 matrix permutations to assess significance. These tests are essentially regression analyses of genetic distance (*F*_ST_, *F*_ST_/(1−*F*_ST_) or *D*_C_) against geographic distance (linear or log-transformed) [63], [64], to determine whether genetic differences show a significant pattern of isolation by distance (IBD). In addition, we also examined the relationship of these same indices of genetic distance with the average number of mitochondrial nucleotide differences between populations (*Π*), obtained with Mega v. 4, considered as a proxy of historical population connectivity [65]. All analyses were bootstrapped over population pairs (10,000 replicates) to generate 95% confidence intervals for *r* ^2^. Geographical distance matrices were generated with GDMG v. 1.2.3 [66].

Using the same two groups of populations, an analysis of molecular variance (AMOVA) in Arlequin v. 3.5 [67] was carried out. Numbers of different alleles between microsatellite haplotypes (amounting to weighted *F*_ST_ over all loci) were used to compute the distance matrix. Significance tests were based on 1,000 permutations.

## Results

### Phylogenetic Analysis of Mitochondrial Haplotypes

The sequences of the two mitochondrial markers (CR and *cytb*) produced essentially similar phylogenetic signals (*P = *0.8149, according to the ILD test), so that they were concatenated to produce a two-gene data set (1,024 bp, containing 162 variable and 75 parsimony informative sites). The null hypothesis of equal evolutionary rate throughout the ML tree was not rejected at a 5% significance level (*P*<0.878), and Bayes factor comparisons rendered very strong evidence in favor of a strict molecular clock (log-difference of 10 units), which was accordingly assumed for all the phylogenetic reconstructions. Two of the samples, *a priori* from *I. monticola*, showed an exceptionally large number of nucleotide differences as compared with the others (mean net *p*-distance = 2.2%; mean *p*-distance among the other *I. monticola* samples = 0.8%). They correspond to individuals from the Sierra de Gistredo, at the SW of the Cantabrian Mountain Range, which is part of those largely independent lesser mountain groups found in the province of León, to the south of the Minho-Sil river basin, and east from the Ancares-Courel groups (see Table S1 and Fig. 1A for geographic details). This region is in-between the known distributions of *I. galani* (to the south) and *I. monticola* (to the northwest). As shown at Figs 1B & S1, the samples from Gistredo form a distinct basal, statistically supported clade (denoted I), whose splitting took place *ca.* 1.6 Million years ago (Mya; 95% confidence interval 1.24–2.00), thus probably meriting the consideration of its full species or subspecies status (see discussion).

**Figure 2:**
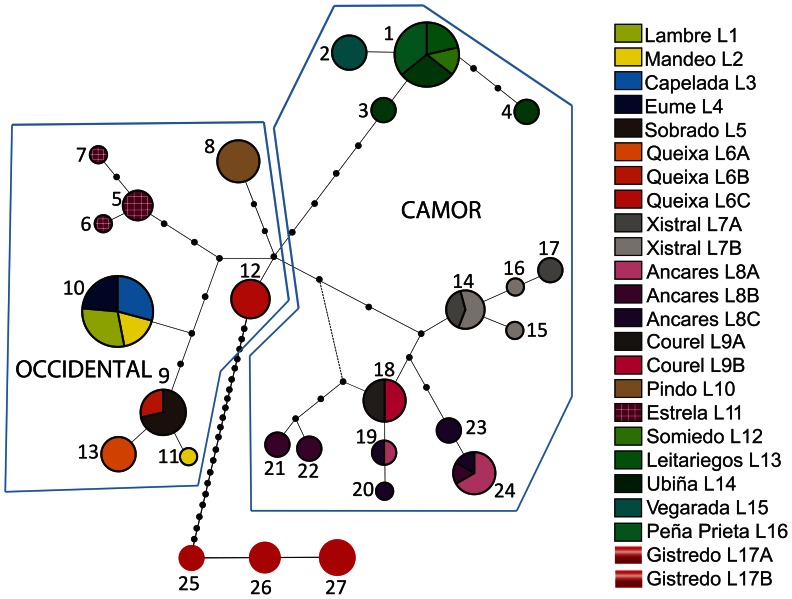
Statistical parsimony network for mtDNA (CR and *cytb*) sequences of *I. monticola*. Circle size reflects the frequency of each haplotype. Small black circles indicate haplotype states that are necessary intermediates but are not present in the sample. Each line represents a single mutational step connecting two haplotypes. CAMOR (Cantabrian Mountain Range) and OCCIDENTAL refer to population assignments based on geographical localization.

As far as *I. monticola* sensu stricto (*i.e.* excluding Gistredo) is concerned, the mitochondrial results indicate that the most recent common ancestor (MRCA) of their different haplotypes dates back to *ca.* 0.9 Mya (0.67–1.25). The samples from the Cantabrian Mountain Range (CAMOR) give rise to two basal haplotype clusters, with strong statistical support, namely Xistral-Courel-Ancares in Galicia, on the one hand (nominally corresponding to the western part of CAMOR; clade III at Fig. 1B), and the rest of the mountain range in León, Asturias and Cantabria (clade II), on the other. Clade III is by far the most rich in haplotype diversity (Fig. 2), particularly at Ancares, where several relatively old mitochondrial lineages coexist at the same sites. It is also worth mentioning, on this respect, that the samples from Xistral constitute a monophyletic group that separated roughly 0.5 Mya (0.27–0.72), and that no matter the method chosen for phylogenetic reconstruction, the single haplotype found at Courel is significantly included in one and the same of the three subclades that subdivide the Ancares sample, whose origin dates back to *ca.* 0.7 Mya (0.43–0.97). The high diversity of the system formed by these mountains stands in sharp contrast with the unexpectedly low diversity and close similarity of the haplotypes observed in the main (eastern) part of the Cantabrian Range (clade II), in spite of the high census size and the large extension occupied by the corresponding populations, without apparent geographic discontinuity among them (Table S1 and Fig. 1A). As for the populations not included in CAMOR, all of them from currently fragmented habitats at the western part of the species range, and collectively denoted as OCCIDENTAL (Fig. 1A), a major clade (V) is observed, which includes the generally only haplotype found in the three riparian habitats (Eume-Lambre-Mandeo) and the low mountains close to them (Capelada), from now on referred to as Artabrian Gulf samples (Fig. 2), in addition to a subclade encompassing Serra de Queixa, Sobrado (lowland) and an haplotype from the Mandeo river basin (Fig. 1B). The MRCA of these sequences dates back to *ca.* 0.4 Mya (0.19–0.62). The single haplotypes found at Pindo and Queixa-1, the two remaining northwestern populations, also began to diverge approximately 0.4 Mya (0.10–0.75). They are joined in a separate clade (VI) both by the MP and the Bayesian reconstruction, but its statistical support is relatively low (Fig. 1B). Marginal model likelihoods show rather strong evidence in favor of this topology, though, when we force this partition to be always present in the sampled trees (log-difference of 2.6 units). Finally, the haplotypes from Serra da Estrela, at the southwest limit of the current distribution of *I. monticola*, form another small clade (IV), which is most closely related to clade V, although the statistical support for this topology (favored by a very strong log-difference of 23 units in Bayes factors comparisons) is not conclusive (see Fig. 1B). Altogether, the topological relationships of clades II, III, IV-V and VI, remain uncertain and give rise to a basal polytomy in the mitochondrial evolution of *I. monticola*, with clear geographic correlates, as shown in Fig. 1.

### Genetic Variation and Equilibrium Tests

A summary of the genetic variation observed at the different microsatellite loci and populations is given at Table S3 and Fig. S2. According to values of *f*_IS_ and the corresponding tests for departure from HW equilibrium, four of the combinations locus x population show a significant excess of homozygotes at the 5% level (after Bonferroni correction), which might be due to segregating null alleles, namely A5 at Queixa-L6A, C113 at Courel-L9A, and PB20 at Courel-L9B and Leitariegos. Applying a conservative criterion, these data were not taken into account for estimating genetic distances among populations, nor for evaluating the evidence of past bottlenecks (see later). However, it should be mentioned that the number of positive *f*_IS_ estimates for each locus and sample nearly doubles the number of negative estimates, and that only two negative global *F*_IS_ scores were obtained for the different samples (see Table S3), thus indicating a general background of homozygote excess, probably associated to population structure (see discussion). Tests of gametic disequilibrium produced no significant result for any pair of loci.

### Identification of Genetically Distinctive Clusters

**Figure 3:**
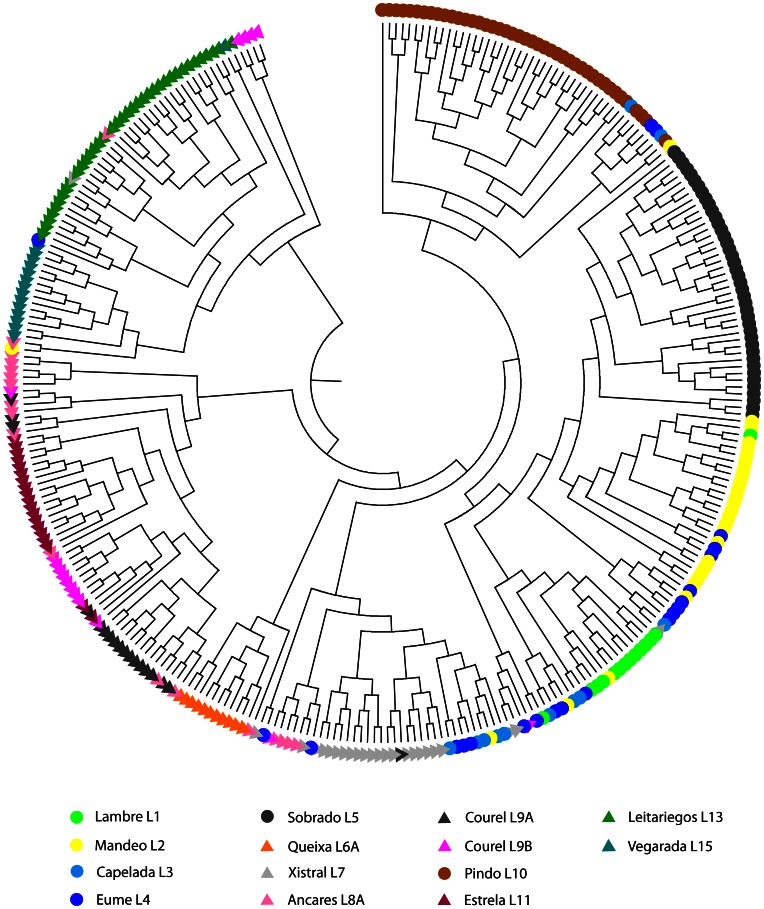
UPGMA tree of collected individuals of *I. monticola* , based on multilocus genotypes, using allele-sharing distances.

**Figure 4:**
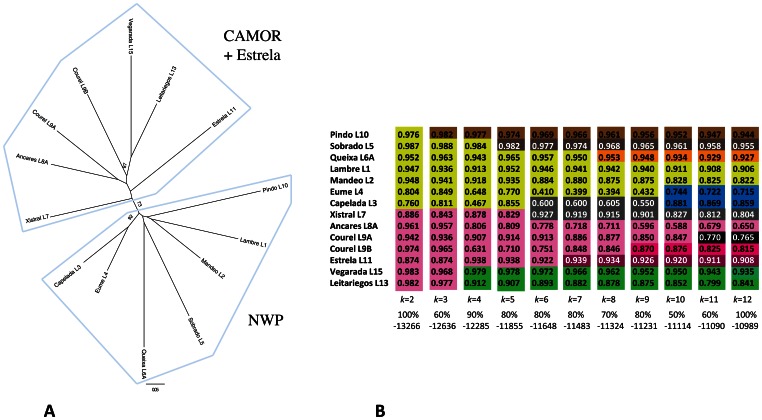
Genetic relatedness among *I. monticola* predefined populations, based on microsatellite multilocus genotypes. **A)** Unrooted NJ tree, using chord distances. Numbers indicate bootstrap support (10,000 samples; only values ≥60% are reported) for the corresponding internal branches. **B)** Assignment of individuals from the predefined populations to *k* clusters, as inferred by STRUCTURE. Colors identify the different clusters inferred by the analysis. Log probabilities of data, *L(k)*, together with % of parallel runs supporting the displayed partition, are shown for each *k*. Abbreviations: CAMOR– Cantabrian Mountain Range; NWP– North Western Populations (*i.e.*, all those included in the occidental group, except Estrela).

**Figure 5:**
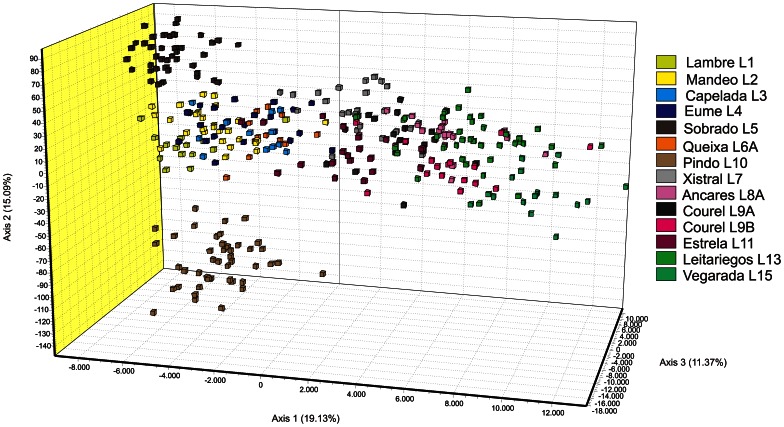
Three-dimensional representation of a Factorial Correspondence Analysis based on microsatellite genotypes of *I. monticola* individuals.

According to Fisher’s exact test, all the sampled populations, except Eume and Capelada, show significantly different gene frequencies at microsatellite loci (*P*<0.0001). On the other hand, individuals from each locality generally group together in a UPGMA tree based on allele-sharing distances (Fig. 3). The reconstruction of the phylogenetic relationships among these populations by the NJ algorithm, using multilocus chord distances, lacks the necessary resolution to obtain significant support for most clusters, though (Fig. 4A). Most important, an internal branch separating CAMOR together with the “occidental” Estrela (CAMOR+E) from the rest of the OCCIDENTAL group (now formed entirely by northwestern populations, NWP), is observed at 73% of the bootstrap samples (see also Table S1 and Fig. 1). Factorial analysis identifies a main axis of variation corresponding to the separation between these same two groups of populations (axis 1 at Fig. 5, explaining 19.13% of total variation), with Estrela and Xistral being the populations of the first group nearest to the second, and a rather good correspondence with the distribution of populations along a longitudinal east-west transect (with negative values for the NWP group, and progressively higher positive values eastwards, for populations within CAMOR+E). The second axis (explaining 15% of total variation) effectively separates three subgroups within the NWP group (namely, Sobrado, Artabrian Gulf+Queixa, and Pindo). Bayesian analysis with Structure unveils a clear hierarchy in the partition of population samples among clusters (Figs 4B & S3), which agrees quite well with these FCA results and the former phylogenetic reconstruction obtained with NJ. The most likely number of clusters is 12, using either *L*(*k*) or Δ*k* as optimization criterion (Fig. S4), which in fact means that each sample is assigned to a genetically differentiated population, with the sole exceptions of Eume-Capelada and Lambre-Mandeo, *i.e.* the Artabrian Gulf samples. At the most basic level (*k = *2, thus forcing the subdivision of samples into two clusters), CAMOR+E and NWP groups become apparent (Figs 4B & S3). It is noteworthy that the individuals from Serra da Estrela are assigned to an exclusive cluster not until *k = *7, being so far grouped together with the samples from Ancares and Courel (see also Fig. 3, for a similar grouping after UPGMA). Generally, more than 70% of the individuals collected at a given locality are assigned to the same cluster, under each of the different levels of structuring examined. The samples from Ancares, on the one hand, and Eume-Capelada, on the other, are exceptional on this respect, since their proportions of membership are sometimes relatively low (Fig. 4B).

### Signatures of Fluctuations in Population Size

As regards the demographic history of these populations, the results of Bottleneck and M-ratio tests are shown in Table 1. Eight of the fourteen statistical populations show heterozygosity excess over mutation-drift expectations, which is highest for Pindo (10.6%) and then for Lambre (5.3%), but not statistically significant in either case. The M-ratio test, however, produced highly significant results for both these populations, as well as for Sobrado, Queixa, Courel-L9B and Vegarada. Using the least restrictive conditions for significance of this test (*i.e.*, comparing observed *m* values with those expected using the value of θ estimated from the corresponding data), all the populations except Eume, Xistral, Ancares, Estrela and Leitariegos, show traces of recent bottlenecks.

### Correlation of Genetic and Geographic Distance Measures

In principle, all CAMOR populations should be connected by gene flow, since no apparent geographical or ecological barrier has been found within their range (see Figure 1A), whereas all the other samples, grouped under the geographic tag of OCCIDENTAL, were obtained from populations nowadays isolated by habitat fragmentation. Independently of this “a prioristic” difference in connectivity, correlations of microsatellite genetic distance with geographical distance (unmodified for potential barriers) were always positive, as expected under a pattern of IBD (see Table 2), although there were clear asymmetries in the performance of the different indices in the two groups of populations. Similar results were obtained in the analysis of association of microsatellite distance with the proxy of historical population connectivity (average number of mitochondrial nucleotide differences between populations, Table 2). The frequent observation of a single and the same haplotype in several of the surveyed populations (see haplotypes 1 and 10, at Fig. 2) precluded IBD analyses using φ_ST_-based distances. This finding, together with the clear geographic subdivision of the genetic diversity of *I. monticola* (and the ensuing reduction of sample sizes), also hindered any attempt to infer the demographic history of its different lineages by mismatch-distribution analyses of pairwise mtDNA differences.

**Table 2:** Analysis of isolation by distance in continuous and fragmented populations of *I. monticola*.

Class of populations	Genetic distance	Connectivity	*r*	*P*-value	*b*
CAMOR	*F*_ST_	*d*	0.871	<0.0001	1.205×10^−6^
		log(*d*)	0.720	<0.0001	0.184
		*Π*	0.546	0.050	0.0118
	*F*_ST_/(1-*F*_ST_)	*d*	0.792	0.0041	1.786×10^−6^
		log(*d*)	0.665	<0.0001	0.272
		*Π*	0.563	0.05	0.0176
	*D*_C_	*d*	0.795	0.0049	1.380×10^−6^
		log(*d*)	0.601	0.0158	0.210
		*Π*	0.408	0.107	0.0136
OCCIDENTAL	*F*_ST_	*d*	0.318	0.2195	6.111×10^−7^
		log(*d*)	0.811	0.0740	0.150
		*Π*	0.445	0.093	0.0269
	*F*_ST_/(1-*F*_ST_)	*d*	0.206	0.2887	9.281×10^−7^
		log(*d*)	0.385	0.1292	0.228
		*Π*	0.337	0.148	0.0408
	*D*_C_	*d*	0.632	0.0055	8.461×10^−7^
		log(*d*)	0.747	0.0088	0.208
		*Π*	0.759	0.003	0.0372

Abbreviations: CAMOR–*a priori* continuous populations from the Cantabrian Mountain Range (L7, L8, L9, L13, L15); OCCIDENTAL–fragmented populations from elsewhere (L1, L2, L3, L4, L5, L6, L10, L11); *F*_ST_ – Weir’s *F*_ST_ genetic distance; *D*_C_–chord genetic distance; *d* – geographic distance between localities, in km; *Π*–average number of mitochondrial nucleotide differences between populations; *r*–estimated correlation coefficient; *P*-value–result of randomization test of significance; *b* – estimated regression coefficient.

### Genetic Consequences of Fragmentation and Reduced Population Size

Results from a two-way AMOVA indicate that most of the observed microsatellite variation is explained by differences within populations (80.5% of total variance, *P*<0.000001). Lower values are related to differences among populations within groups (14.5% of total variance, *P*<0.000001), and still lower to differences among groups (CAMOR *vs.* OCCIDENTAL, 5.0% of total variance, *P = *0.00391). Results from several one-way AMOVAs, together with different measures of genetic diversity in continuous and fragmented populations are shown in Table 3. While both of these groups harbor similar levels of genetic variation at microsatellite loci, the among-populations component is estimated to be higher at OCCIDENTAL (18%, as compared with 11% at CAMOR), but the difference is not statistically significant. As for genetic diversity, all the scores are lower for OCCIDENTAL, both in terms of mean number of alleles per locus and mean heterozigosity within populations (see Table 4). However, as shown in this same table, all these observations arise not from the fragmented condition of these populations, but from the reduced size of most of them. Variation among fragmented populations of reduced size (OCCIDENTAL_R) is 100% higher than among continuous populations, as measured by the *F*_ST_ score (0.215 *vs.* 0.107), whereas the mean number of alleles per locus and expected heterozygosis at HWE are considerably lower in them (by a 34% and a 18%, respectively). Actually, OCCIDENTAL_R shows lower average numbers of alleles in 10 out of the 11 loci analyzed, and lower heterozygosis in nine of them (Table 4; *P* = 0.0024 and *P* = 0.0068, respectively, after Wilcoxon signed rank-tests). In addition, on average, large populations show an excess of homozygotes over HW expectations in a significant proportion of loci (*P* = 0.0087, one-tail Wilcoxon signed rank-test); this observation is consistent with mean *F*_IS_ scores for these categories, which can be easily obtained from Table 1, namely 0.018 and 0.060 for reduced and large populations, respectively). Finally, *θ* estimates derived from mean expected heterozygosis point out a reduction of 48% in the scores obtained from reduced populations (1.62), as compared with large populations (3.10), which, assuming equal mutation rates in both classes, amounts to an equivalent descent in their corresponding mean effective population sizes.

**Table 3:** AMOVA and diversity indices in continuous (CAMOR) *vs.* fragmented (OCCIDENTAL) populations of *I. monticola*, based on microsatellite variation.

Class of populations	Variance components (d. f.)		*F*_ST_-like	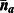 (s. e.)	*H*_HW_ (s. e.)	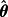
	Among	Within				
CAMOR	0.496 (5)	4.148 (250)	0.107	7.1 (0.58)	0.753 (0.0270)	3.05
OCCIDENTAL	0.802 (7)	3.545 (368)	0.184	5.4 (0.39)	0.666 (0.0245)	1.99
OCCIDENTAL_L	0.494 (2)	4.075 (99)	0.108	6.7 (0.50)	0.766 (0.0200)	3.27
OCCIDENTAL_R	0.917 (4)	3.35 (269)	0.215	4.7 (0.43)	0.619 (0.0342)	1.62

Abbreviations: CAMOR–*a priori* continuous populations from the Cantabrian Mountain Range; OCCIDENTAL–fragmented populations from elsewhere; OCCIDENTAL_L–fragmented populations of large size (L3, L4, L11); OCCIDENTAL_R–fragmented populations of reduced size (L1, L2, L5, L6, L10); d. f. – degrees of freedom; – observed mean number of alleles; *H*_HW_ – expected heterozygosity at Hardy-Weinberg equilibrium; s. e. – standard error; 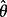
–estimate of the population mutational parameter.

**Table 4:** Indices of genetic diversity at microsatellite loci in continuous and fragmented populations of *I. monticola.*

Class of populations	Geneticindex	Microsatellite loci											Average
		C118	C103	D115	B107	C113	C9	B135	A5	Pb22	Pb26	Ay26	
CAMOR	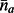	5.8	6.3	7.7	7.0	6.0	7.3	6.7	8	7.3	4.0	11.8	7.1
	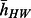	0.677	0.760	0.832	0.797	0.695	0.806	0.793	0.700	0.793	0.556	0.877	0.753
	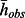	0.650	0.731	0.770	0.742	0.515	0.807	0.723	0.633	0.555	0.628	0.829	0.689
OCCIDENTAL_L	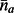	4.7	6.7	10.3	7.0	4.7	5.0	7.0	7.0	8.3	6.0	6.7	6.7
	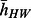	0.618	0.792	0.888	0.776	0.724	0.779	0.786	0.734	0.827	0.740	0.763	0.766
	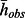	0.591	0.777	0.823	0.675	0.696	0.818	0.731	0.477	0.806	0.749	0.795	0.722
OCCIDENTAL_R	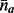	4.0	4.0	6.8	3.0	4.8	4.8	3.4	4.2	4.6	4.2	7.8	4.7
	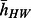	0.621	0.507	0.774	0.383	0.696	0.685	0.623	0.529	0.582	0.662	0.743	0.619
	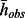	0.672	0.476	0.816	0.380	0.563	0.703	0.636	0.355	0.558	0.670	0.751	0.598

Abbreviations: Population groups as in Table 4; 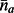
– mean within-class observed number of alleles; 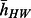
– mean within-class expected frequency of heterozygotes at Hardy-Weinberg equilibrium; 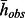
– mean within-class observed frequency of heterozygotes.

## Discussion

### Iberian Rock-lizards Speciation

Comparative phylogeographic analyses have shown strong genetic subdivisions, indicative of extended periods of population isolation, for many species and species complexes in the Iberian Peninsula, and especially for those with limited effective dispersal that live in mountains. This is most likely due to their survival throughout the Pleistocene ice ages in multiple refugia or sanctuaries, which tend to occur in mountain ranges [68], [69]. Iberian rock-lizards (*Iberolacerta* spp.; phylogenetic revision in [12] and [10]) offer some good examples of this evolutionary pattern [11]–[14], [70]. Two recently described species, namely *I. martinezricai* (see [23], [71]) and *I. galani* [10], have a restricted montane distribution not far away from *I. monticola* populations (see Fig. 1). But the list of *Iberolacerta* species or subspecies in this region may not be exhausted. The denomination of Montes de León brings together a number of largely independent mountain groups, which constitute a perfect scenario for the emergence of parapatric genetic lineages. The mitochondrial results from the Sierra de Gistredo offer the first evidence on this respect, pointing out the existence of a clade clearly distinct from the three other species, whose independent evolution began roughly 1.6 Mya, at Early Pleistocene. Additional evidence from morphology (biometry and scalation), karyology and osteology, will be necessary to establish the definitive taxonomic status of this population, together with microsatellite data to investigate its genetic structure (in preparation).

### Phylogeography of *Iberolacerta monticola* Populations

The mitochondrial results are consistent with the simultaneous vicariance of not less than four, and a maximum of six populations after the mid-Pleistocene revolution (about 0.9 Mya), one of the two major climate transitions of the Quaternary period, with drastic effects on the geographical distributions of many species [72]–[74]. In fact, several of the modern populations of the species reside in areas that have been suggested to serve as glacial refugia, on the basis of the distribution of many different plant and animal species (namely Serra da Estrela, and the western and eastern parts of the Cantabrian Mountain Range [68], [75]). Thus, the often-cited subdivision of *I. monticola* into *I. m. monticola* at Serra da Estrela, and *I. m. cantabrica* elsewhere, falls too short to describe the actual pattern of diversification within this species. With the conspicuous exception of Serra de Ancares, which shows extensive mitochondrial admixture, most other populations are characterized by parapatrically distributed clades and subclades. According to their estimated coalescence times, this pattern could be the outcome of the relatively recent (re)colonization of these areas from different refugial populations, which, as in other well documented cases [76], [77], have persisted without admixture during several ice ages. So the partition of Ancares-Courel *vs.* Xistral (clade III) in the CAMOR group, or the low-altitude Artabrian Gulf *vs.* the moderate-high altitude populations of Serra da Queixa (clade V) in the OCCIDENTAL group, whose dating (mean node ages of 0.4–0.5 Mya) agrees quite well with the second distinct climate change of the Quaternary, the mid-Bruhnes event, about 0.43 Mya [78].

The primarily east-west orientation of the Cantabrian Range may have facilitated the survival of populations simply by moving up or down mountains, as the general climate worsens or ameliorates [79]. In this scenario, the model of “refugia within refugia”, put forward to explain the phylogeographic patterns observed for a range of fauna and flora in the Iberian Peninsula [68], offers a good frame for all our findings. In general, deeper intraspecific lineages are expected in populations whose range included several glacial refugia (that could be Ancares), whereas shallower and relatively impoverished lineages are expected for populations resulting from recent recolonization events (as found elsewhere). The impoverished mitochondrial diversity observed in the eastern clade (II), which includes the most widespread and largest *I. monticola* populations, might also be explained by a recent selective sweep, as suggested in other cases [80], [81]. But even so, the relatively old origin of this clade would demand an explanation involving the interaction of selection and gene flow [82]. Fine-scale analyses of the putative hybrid zones, involving nuclear markers (see [83], [84] for a similar case in *Lacerta schreiberi*), will be necessary for a more thorough understanding of the evolution of these populations, which undoubtedly have experienced multiple episodes of admixture.

On general grounds, perhaps the main factor determining the level of genetic and regional subdivision of species inhabiting European mountains is the duration of the cycles of range contraction and expansion triggered by climate changes. For species dwelling at upper elevation (alpine) habitats, vicariance events are most likely associated to warm, relatively short interglacials, thus giving rise to shallow phylogenies. On the contrary, according to the displacement refugia model [3], species adapted to lower habitats are predicted to show stronger phylogeographic signals, due to prolonged fragmentation during the long glacial periods. The Pyrenean rock lizard *Iberolacerta bonnali*, which inhabits rocky habitats at 1,600–3,300 m asl, and whose current distribution is most likely a consequence of post-glacial range fragmentation after the end of the Last Glacial Maximum, would perfectly fit into the first category [70]. *Iberolacerta monticola* should be expected to lie somewhere in-between the two extremes. Its mitochondrial lineages seem to have Pleistocene origins, with diversification times estimated to be younger than 1.5 Mya, in contrast to other Iberian species characterized by the persistence of generally much older lineages through glacial cycles [85], [86], which also seem to fit into the “refugia within refugia” model. This difference is simply a consequence of current taxonomic boundaries: older mitochondrial lineages of *Iberolacerta* do in fact exist, but they are nowadays associated to different, closely related species. In terms of the phylogeographic models of “S” (sanctuary) and “R” (refugia) species [69], the shallow mitochondrial diversification obtained from most clades of *I. monticola* (a truly, but relatively young “S” species, which apparently originated somewhere in this same region that it now inhabits, and shows different alleles that bear testimony of their ancestral diversity) is actually similar to the genetic signature of “R” species (taxa that colonized Iberia during the Quaternary, and lost a major part of their older lineages after extinction in all ancestral territories due to climate change).

### Loss of Atlantic Forests and Habitat Fragmentation

In addition to the large effects of glaciations on the diversification of this species, the combined evidence from microsatellites and mitochondrial markers clearly shows that its current fragmented distribution at most nearby lowland populations has a very recent origin. All the species of *Iberolacerta*, except *I. monticola*, are confined to mountains in the centre and NE of the Iberian Peninsula, probably because of the low thermal quality and scarcity of refuges of lower elevation environments at those regions [87]. In the NW, the observation of lowland populations associated to relict Atlantic forests of an otherwise montane species is not an exclusive characteristic of *I. monticola* [88]–[92]. Major woodland declines in NW Iberia occurred during the Galician-Roman Medieval Period [93], [94], often due to recurrent fires produced by past human societies to facilitate grazing [95]. The loss of intervening suitable habitat, and the orientation of the retreating front of the Atlantic forests (from west to east), should have produced a succession of splits of the gene pools of *I. monticola* populations, which can be traced through the results of all our analyses of microsatellite variation, but particularly of FCA and Mantel tests. The significant association of Cavalli-Sforza chord distance (*D*_C_), but not *F*_ST_-based indices, both with connectivity and with log-distance between populations, probably reflects the better performance of *D*_C_ in recovering the topology of microsatellite trees [39], [64], notwithstanding the fact that most internal branches of the NJ tree for *I. monticola* populations have low statistical support. This still on-going process of fragmentation has produced a patchy distribution of *I. monticola* in the NW of the Iberian Peninsula, which includes large, moderate and small sized populations, and both montane and lowland habitats (OCCIDENTAL group).

### Conflicting Phylogenetic Signals from Nuclear and Mitochondrial Markers at the Population of Serra da Estrela

Although mtDNA data suggest that the Estrela population is most closely related to other occidental populations (at Serra da Queixa and the Artabrian Gulf), thus implying that it has been separated from the CAMOR populations for nearly 1 million years, the microsatellite data do not support this conclusion, and indicate that it has been last connected with the populations of Courel and Ancares, at the western part of the Cantabrian Range, instead. Sex-biased gene flow is the most common explanation for such discordant patterns of biparental nuclear and maternal mitochondrial markers [96], but the evidence for higher male gene flow in *I. monticola* is so far not conclusive, and mostly based on differences in home-range sizes of the closely related species *I. cyreni* [97], [98]. However, male-biased dispersal is a quite likely characteristic for most lizards with polyginous mating systems [99], such as *I. monticola*, so that it is probably a contributing factor to the phylogenetic pattern observed at Estrela. But there are other, not mutually excluding possibilities as well. One plausible scenario is that after the few hundred thousand years that followed the vicariance episodes, during which the different mitochondrial lineages of *I. monticola* evolved in isolation, there would have been a colonization of Serra da Estrela from northeastern populations (Courel-Ancares). A mitochondrial “occidental” allele may have drifted by chance to high frequency at the population of Estrela, even though the nuclear genomes bear the trace of that secondary contact. If the above scenario is correct then there may be alleles from the Cantabrian lineage III still present elsewhere in the Serra da Estrela.

### Genetic Signatures of Isolation and Population Size Reduction

Small isolated populations of *I. monticola* show footprints of genetic erosion, such as a reduction of standing levels of genetic variation and, insofar as *F*_ST_ is accepted as a valid index of drift load [100], [101], a relative increase in the expected frequency of fixed deleterious mutations. Taking into account that endangered populations of other species generally display *F*_ST_ values exceeding 0.2 [64], [102], [103], then the conclusion that *I. monticola* is threatened by extinction in these isolated sites seems justified, particularly so if considering that there has been scarcely time for these populations to reach mutation-drift equilibrium, so that the observed *F_ST_* value of 0.215 (twice the value obtained for “continuous” populations) is most likely to be an underestimate. However, there are other considerations that must be taken into account on this respect.

First, the frequency of segregating deleterious mutations and the mutation load of *I. monticola* at pre-fragmentation stages may have been much lower than for an undivided population. It all depends on whether the species is expected to largely fit a metapopulation model or not. In species displaying low natal and breeding dispersal, such as many lizards [104], [105], individuals are more likely to mate with those nearby, so that populations distributed over continuous habitats may actually be considered as large metapopulations, with fewer deleterious alleles and potentially a lower mutation load than an undivided population [106]. Even for modest *F*_ST_ scores, such as those observed among “continuous” populations of *I. monticola*, the expected reduction in inbreeding depression and mutation load of the metapopulation as compared with an undivided population can be quite important [107], [108], thus being less prone to experience negative genetic effects upon fragmentation.

Second, small isolates of *I. monticola* show no evidence of overall excess of homozygotes, not even Lambre, a very small population that may have gone extinct by now [19], although inbreeding should be, in principle, much more intense in them than in large populations. This could be due, at least in part, to transient associative overdominance, produced either by local genetic linkage to the target loci of selection [109] or genomic effects on fitness [110]. Besides, females may be choosing to mate with unrelated males in these reduced populations, or there may be overdispersion with respect to genetic similarity, similarly to what has been observed or suggested for other species after population declines in a variety of evolutionary contexts [111]–[116].

### Concluding Remarks

The tempo and mode of the building-up of genetic differences among populations that ultimately become new species may assume *endless forms* [117]. However, many species and species complexes in the Iberian Peninsula show concordant phylogeographic patterns, produced by the strong differentiation of populations that have survived in separate refugia throughout Pleistocene ice ages, aided by the high physiographic complexity and the wide range of climates of this part of the world [68]. The persistence of these geographically congruent differentiated lineages in spite of potential gene flow, when cyclic range expansions presumably bring many of them repeatedly into contact, suggests the involvement of a general mechanism of speciation mediated by the evolution of intrinsic postzygotic isolating barriers [118]. The test of this hypothesis should await for studies on the distribution of genotypes over a very small spatial scale at suture zones, complemented by fitness analyses of natural or experimental hybrids.

The Holocene may represent an inflexion point for the long-term survival of those genetic lineages more exposed to the anthropogenic perturbations of their habitats. Thus, successful heirs of many thousand years of evolution, with unique genetic characteristics forged in the engine of the cyclic climate changes of the Pleistocene, might not get through the next glacial stage, but become extinct relatively soon.

## Supporting Information

Figure S1 **Bayesian phylogenetic tree of** ***I. monticola*** **populations based on mitochondrial sequences.** The tree is rooted using *I. cyreni* as outgroup, and it includes representative sequences from *I. martinezricai* and *I. galani*. See legend of Figure 1B for instructions to read the statistical support of internal branches.
(DOC)

Figure S2 **Different estimates of genetic variation at microsatellite loci, for the different geographical samples of** ***I. monticola.*** **(A)** Expected heterozygosity in HW equilibrium (He); bars correspond to standard deviations. **(B)** Average allelic richness (blue bars) and private allelic richness (red bars), corrected for sample size.
(DOC)

Figure S3 **Results of the analysis for genetic clustering using Structure.** Colors correspond to the different clusters inferred by the analysis. The log likelihood of each assumed number of clusters (ln*L*) is shown, together with the % of independent runs that correspond to the represented partition of populations among clusters.
(DOC)

Figure S4 **Identification of the most likely number of** ***I. monticola*** **populations by the analysis of microsatellite data with Structurev. 2.1**. **(A)** Estimated log probability of data for the different number of inferred clusters (*K*); bars correspond to standard deviation, after 10 independent runs. **(B)** Rate of change in the log probability of data between successive *k* values (Δ*k*). Both figures were obtained with the aid of Structure Harvester v. 0.56.4, http://taylor0.biology.ucla.edu/struct_harvest/.
(DOC)

Table S1 **Information about the populations and the genetic samples of** ***I. monticola*** **used in this work.**
(DOC)

Table S2 **Information summary of the DNA markers used in this work.**
(DOC)

Table S3 **Genetic variation at microsatellite loci in populations of** ***I. monticola*** **(original data without any correction).**
(DOC)
