# Supplementary material for: Causes and Evolutionary Consequences of Population Subdivision of an Iberian Mountain Lizard, Iberolacerta monticola
Source: PLoS One. 2013 Jun 7;8(6):e66034. doi: 10.1371/journal.pone.0066034 (PMC3676366; doi:10.1371/journal.pone.0066034)

**Figure S1. Bayesian phylogenetic tree of *I. monticola* populations based on mitochondrial sequences.** The tree is rooted using *I. cyreni* as outgroup, and it includes representative sequences from *I. martinezricai* and *I. galani*. The statistical support of internal branches is indicated as in Figure 1B.


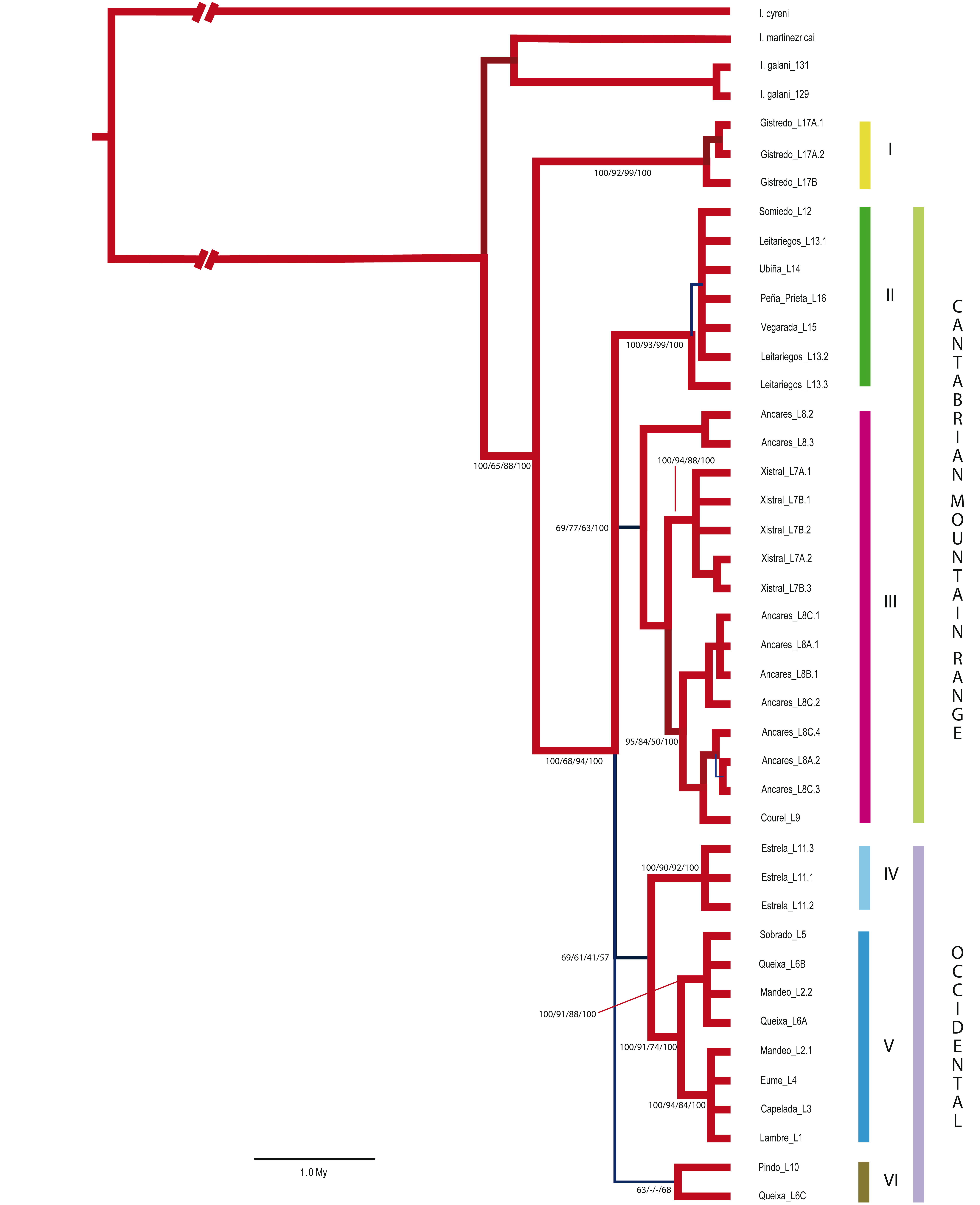

Supplement: Figure S1 — Bayesian phylogenetic tree of I. monticola populations based on mitochondrial sequences. The tree is rooted using I. cyreni as outgroup, and it includes representative sequences from I. martinezricai and I. galani. See legend of Figure 1B for instructions to read the statistical support of internal branches. (DOC) [file pone.0066034.s001.doc]
