# Supplementary material for: Causes and Evolutionary Consequences of Population Subdivision of an Iberian Mountain Lizard, Iberolacerta monticola
Source: PLoS One. 2013 Jun 7;8(6):e66034. doi: 10.1371/journal.pone.0066034 (PMC3676366; doi:10.1371/journal.pone.0066034)
Supplement: Figure S2 — Different estimates of genetic variation at microsatellite loci, for the different geographical samples of I. monticola. (A) Expected heterozygosity in HW equilibrium (He); bars correspond to standard deviations. (B) Average allelic richness (blue bars) and private allelic richness (red bars), corrected for sample size. (DOC) [file pone.0066034.s002.doc]

**Figure S2. Different estimates of genetic variation at microsatellite loci, for the different geographical samples of *I. monticola.*** **(A)** Expected heterozygosity in HW equilibrium (He); bars correspond to standard deviations. **(B)** Average allelic richness (blue bars) and private allelic richness (red bars), corrected for sample size.

**A**


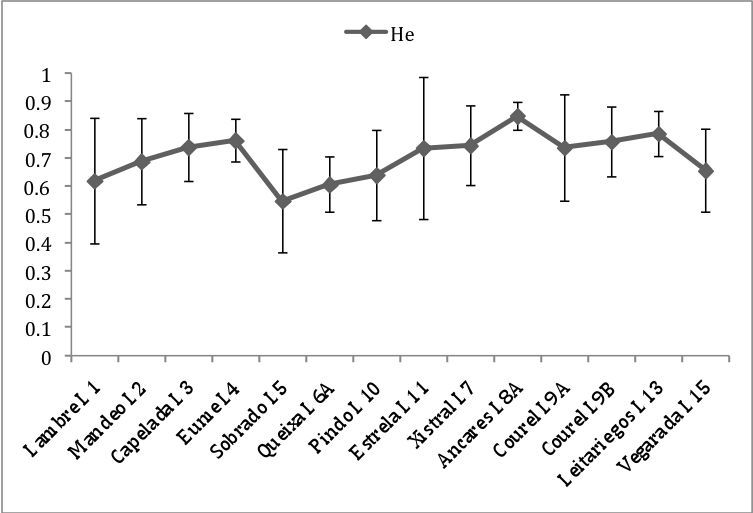


**B**

**
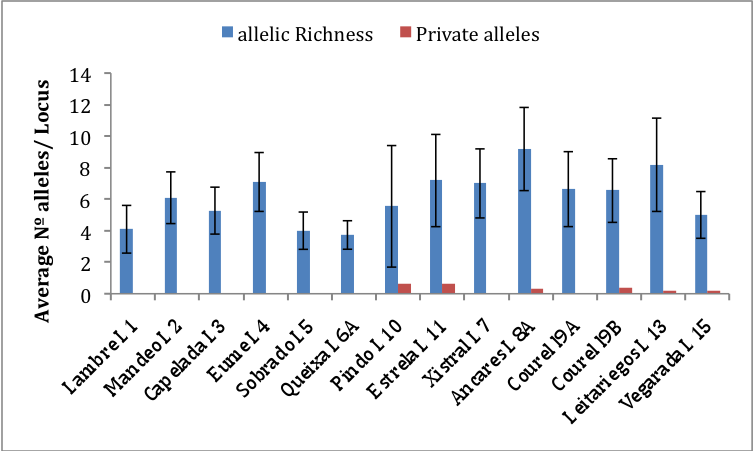
**
