# Supplementary material for: Causes and Evolutionary Consequences of Population Subdivision of an Iberian Mountain Lizard, Iberolacerta monticola
Source: PLoS One. 2013 Jun 7;8(6):e66034. doi: 10.1371/journal.pone.0066034 (PMC3676366; doi:10.1371/journal.pone.0066034)

**Figure S3. Results of the analysis for genetic clustering using Structure.** Colors correspond to the different clusters inferred by the analysis. The log likelihood of each assumed number of clusters (ln*L*) is shown, together with the % of independent runs that correspond to the represented partition of populations among clusters.


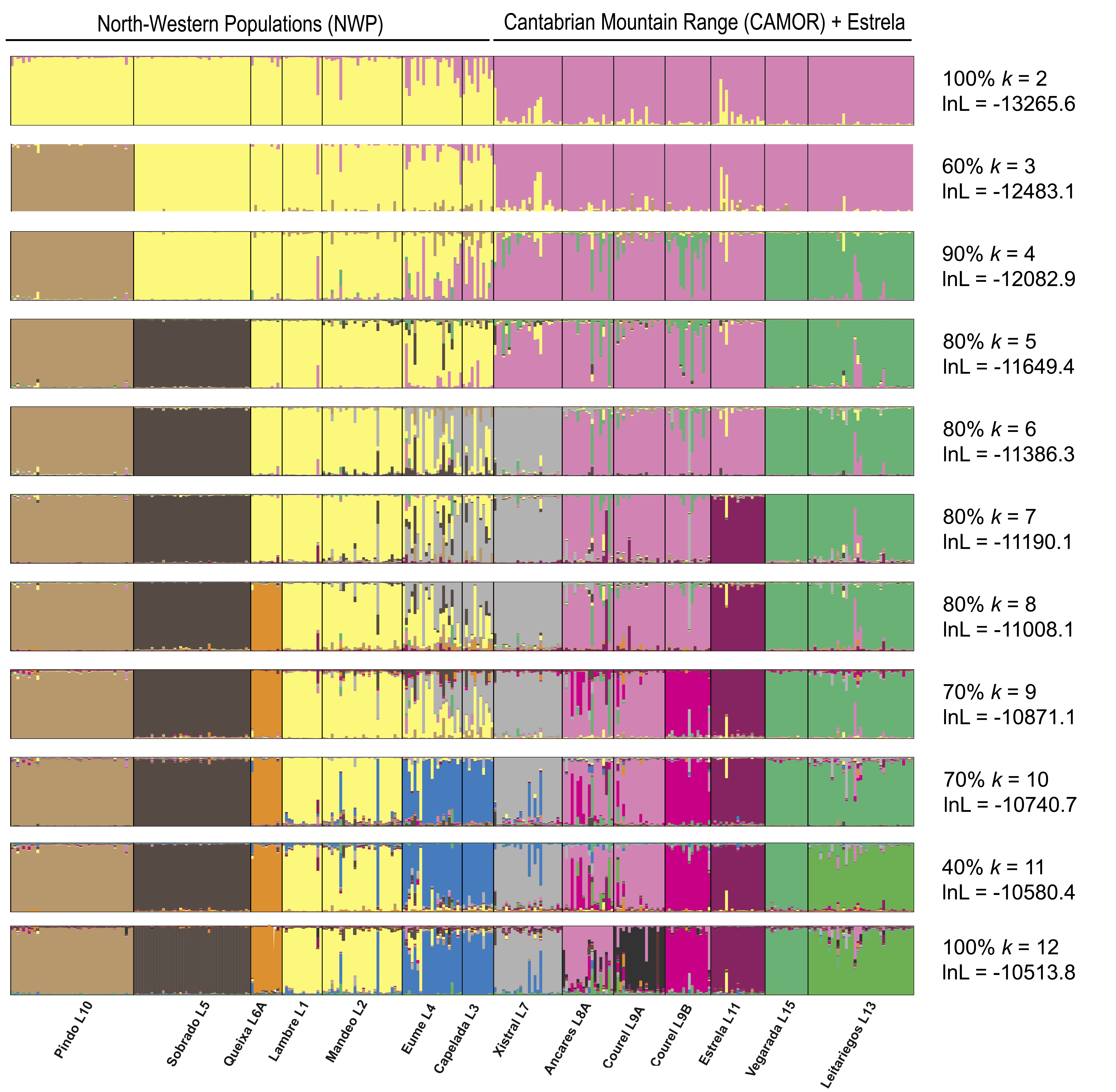

Supplement: Figure S3 — Results of the analysis for genetic clustering using Structure. Colors correspond to the different clusters inferred by the analysis. The log likelihood of each assumed number of clusters (lnL) is shown, together with the % of independent runs that correspond to the represented partition of populations among clusters. (DOC) [file pone.0066034.s003.doc]
