# Supplementary material for: Causes and Evolutionary Consequences of Population Subdivision of an Iberian Mountain Lizard, Iberolacerta monticola
Source: PLoS One. 2013 Jun 7;8(6):e66034. doi: 10.1371/journal.pone.0066034 (PMC3676366; doi:10.1371/journal.pone.0066034)
Supplement: Figure S4 — Identification of the most likely number of I. monticola populations by the analysis of microsatellite data with Structurev. 2.1. (A) Estimated log probability of data for the different number of inferred clusters (K); bars correspond to standard deviation, after 10 independent runs. (B) Rate of change in the log probability of data between successive k values (Δk). Both figures were obtained with the aid of Structure Harvester v. 0.56.4, http://taylor0.biology.ucla.edu/struct_harvest/. (DOC) [file pone.0066034.s004.doc]

**Figure S4. Identification of the most likely number of *I. monticola* populations by the analysis of microsatellite data with Structure v. 2.1**. **(A)** Estimated log probability of data for the different number of inferred clusters (*K*); bars correspond to standard deviation, after 10 independent runs. **(B)** Rate of change in the log probability of data between successive *k* values (Δ*k*). Both figures were obtained with the aid of Structure Harvester v. 0.56.4, <http://taylor0.biology.ucla.edu/struct_harvest/>.

**A**


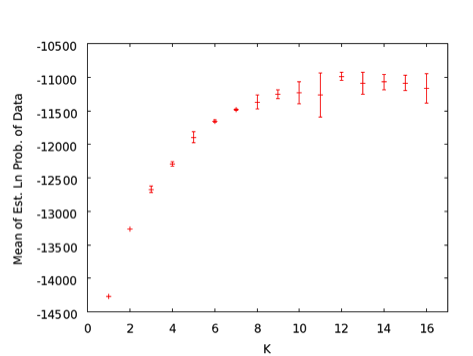


**B**

**
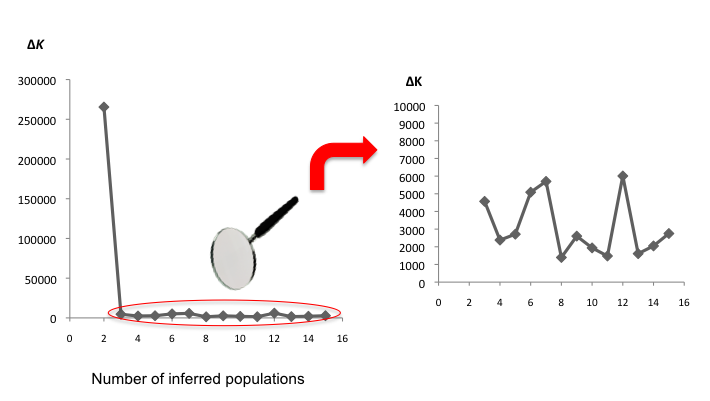
**
