# Supplementary material for: Causes and Evolutionary Consequences of Population Subdivision of an Iberian Mountain Lizard, Iberolacerta monticola
Source: PLoS One. 2013 Jun 7;8(6):e66034. doi: 10.1371/journal.pone.0066034 (PMC3676366; doi:10.1371/journal.pone.0066034)
Supplement: Table S1 — Information about the populations and the genetic samples of I. monticola used in this work. (DOC) [file pone.0066034.s005.doc]

| **Label** | **Site** | **Population** | **Location** | | |  | **Sample size** | | |  | **GenBank acc. nos.** | |  | **Haplotype** |
| --- | --- | --- | --- | --- | --- | --- | --- | --- | --- | --- | --- | --- | --- | --- |
|  |  | **size** | **lat** | **lon** | **alt** |  | **msat** | **CR** | ***cytb*** |  | **CR** | ***cytb*** |  | **labels** |
| L1 | Lambre | * | 43.31 | -8.14 | 35 |  | 14 | 6 | 5 |  | EF121828 | HQ234883 |  | 10 |
| L2 | Mandeo | ** | 43.26 | -8.13 | 30-90 |  | 28 | 5 | 4 |  | EF121828 | HQ234883-84 |  | 10, 11 |
| L3 | Capelada (Serra da) | *** | 43.70 | -7.96 | 150-450 |  | 11 | 5 | 5 |  | EF121828 | HQ234883 |  | 10 |
| L4 | Eume | *** | 43.41 | -8.07 | 20-300 |  | 21 | 5 | 4 |  | EF121828 | HQ234883 |  | 10 |
| L5 | Sobrado | ** | 43.04 | -8.02 | 511 |  | 41 | 5 | 5 |  | EF121828 | HQ234885 |  | 9 |
| L6A | Queixa (Serra da) | ** | 42.25 | -7.30 | 1750 |  | 11 | 5 | 4 |  | EF121829 | HQ234885 |  | 13 |
| L6B |  | ** | 42.25 | -7.37 | 920 |  | - | 2 | 2 |  | EF121828 | HQ234885 |  | 9 |
| L6C |  | * | 42.27 | -7.35 | 1000 |  | - | 8 | 7 |  | EF121827 | HQ234886 |  | 12 |
| L7A | Xistral (Serra do) | *** | 43.36 | -7.46 | 600 |  | 6 | 5 | 4 |  | EF121827 | HQ234887-88 |  | 14, 17 |
| L7B |  | *** | 43.49 | -7.60 | 700 |  | 18 | 5 | 5 |  | EF121827 | HQ234887  HQ234889-90 |  | 14, 15, 16 |
| L8A | Ancares (Serra dos) | *** | 42.85 | -6.87 | 1100 |  | 18 | 7 | 5 |  | EF121828  EF121831 | AY267235  AY267236 |  | 19, 24 |
| L8B |  | *** | 42.86 | -6.81 | 1650 |  | - | 13 | 8 |  | EF121828  EF121831 | AY267235  HQ234891-92 |  | 21, 22, 24 |
| L8C |  | *** | 42.84 | -6.83 | 1350 |  | - | 5 | 5 |  | EF121828  EF121831 | AY267235-36  HQ234893-94 |  | 19, 20, 23, 24 |
| L9A | Courel (Serra do) | *** | 42.62 | -7.22 | 790 |  | 18 | 5 | 3 |  | EF121831 | HQ234895 |  | 18 |
| L9B |  | *** | 42.66 | -7.34 | 700 |  | 16 | 7 | 3 |  | EF121831 | HQ234895 |  | 18 |
| L10 | Pindo (Montes do) | ** | 42.88 | -9.11 | 340–629 |  | 43 | 10 | 6 |  | EF121830 | HQ234896 |  | 8 |
| L11 | Estrela (Serra da) | *** | 40.32 | -7.61 | 1800 |  | 19 | 12 | 6 |  | AY267247 | JN048498-99  GQ142124 |  | 5, 6, 7 |
| L12 | Somiedo (CMR) | *** | 43.01 | -5.57 | 1600 |  | - | 5 | 3 |  | EF121832 | HQ234897 |  | 1 |
| L13 | Leitariegos (CMR) | *** | 42.99 | -6.41 | 1530 |  | 37 | 6 | 6 |  | EF121832  HQ234880 | HQ234897-98 |  | 1, 3, 4 |
| L14 | Ubiña (CMR) | *** | 42.59 | -6.24 | 1800 |  | - | 4 | 4 |  | EF121832 | HQ234897 |  | 1 |
| L15 | Vegarada (CMR) | *** | 43.04 | -5.46 | 1560 |  | 15 | 5 | 4 |  | EF121832 | HQ234899 |  | 2 |
| L16 | Peña Prieta (CMR) | *** | 43.02 | -4.72 | 2050 |  | - | 5 | 5 |  | EF121832 | HQ234897 |  | 1 |
| L17A | Gistredo (Sierra de) | *** | 42.85 | -6.28 | 1600 |  | - | 7 | 7 |  | HQ234877-78 | HQ234900 |  | 26, 27 |
| L17B |  | *** | 42.90 | -6.19 | 1433 |  | - | 2 | 2 |  | HQ234879 | HQ234900 |  | 25 |

**Table S1**. **Information about the populations and the genetic samples of *I. monticola* used in this work.**

Sample sizes include 17 individuals for the CR and 6 individuals for *cytb* reported by Crochet *et al.* (2004).

Abbreviations: lat  latitude; lon  longitude; alt  altitude (meters above sea level); msat  microsatellites; CR  control region; *cytb*  cytochrome *b*; CMRCantabrian Mountain Range. Site-labels identify sampling locations at Fig. 1 and throughout the main text. *very small population (<30 individuals); ** population of small or moderate size (500–10,000 individuals); *** large population (>10,000 individuals).
