# Supplementary material for: Causes and Evolutionary Consequences of Population Subdivision of an Iberian Mountain Lizard, Iberolacerta monticola
Source: PLoS One. 2013 Jun 7;8(6):e66034. doi: 10.1371/journal.pone.0066034 (PMC3676366; doi:10.1371/journal.pone.0066034)
Supplement: Table S2 — Information summary of the DNA markers used in this work. (DOC) [file pone.0066034.s006.doc]

**Table S2. Information summary of the DNA markers used in this work.**

| **Reaction** | **Accession No.** | **Tag** | **Tm** | **References** |
| --- | --- | --- | --- | --- |
| Multiplex 1 | AY545222 | Pb22 | 53ºC | [1] |
|  | AY545226 | Pb26 |  |  |
| Multiplex 2 | AY147826 | Ay26 | 52ºC | [2] |
|  | EU233464 | B135 |  | [3] |
| Multiplex 3 | EU233469 | C118 | 53ºC | [3] |
|  | EU233467 | C103 |  |  |
| Multiplex 4 | EU233462 | B114 | 53ºC | [3] |
|  | EU233472 | D115 |  |  |
| Multiplex 5 | EU233461 | B107 | 53ºC | [3] |
|  | EU233468 | C113 |  |  |
| Cytochrome *b*^1^ | AM176577 | *cytb* | 51ºC | [4] |
| Control Region^2^ | AM176577 | CR | 50ºC | [4] |

**^1,2^** Part of the cytochrome *b* gene (*cytb*, 598 bp) and part of the control region (CR, 454 bp), corresponding to position intervals 14,169–14,763 and 16,310–16,769, respectively, of the *Lacerta viridis* mitochondrial genome (GenBank acc. no. AM176577).

**REFERENCES**

1 Pinho C, Sequeira F, Godinho R, Harris DJ, Ferrand N (2004) Isolation and characterization of nine microsatellite loci in *Podarcis bocagei* (Squamata : Lacertidae). Mol Ecol Notes 4: 286-288.

2 Nembrini M, Oppliger A (2003) Characterization of microsatellite loci in the wall lizard *Podarcis muralis* (Sauria : Lacertidae). Mol Ecol Notes 3: 123-124.

3 Remón N, Vila M, Galán P, Naveira H (2008) Isolation and characterization of polymorphic microsatellite markers in I*berolacerta monticola*, and cross-species amplification in *Iberolacerta galani* and *Zootoca vivipara.* Mol Ecol Resour 8: 1351-1353.

4 Crochet PA, Chaline O, Surget-Groba Y, Debain C, Cheylan M (2004) Speciation in mountains: phylogeography and phylogeny of the rock lizards genus *Iberolacerta* (Reptilia: Lacertidae). Mol Phylogenet Evol 30: 860-866.
