# Supplementary material for: Causes and Evolutionary Consequences of Population Subdivision of an Iberian Mountain Lizard, Iberolacerta monticola
Source: PLoS One. 2013 Jun 7;8(6):e66034. doi: 10.1371/journal.pone.0066034 (PMC3676366; doi:10.1371/journal.pone.0066034)
Supplement: Table S3 — Genetic variation at microsatellite loci in populations of I. monticola (original data without any correction). (DOC) [file pone.0066034.s007.doc]

**Table S3**. **Genetic variation in populations of *I. monticola* (original data without any correction).**

| **All loci** | | | | | |  | ***f*_IS_, *n*_a_ and *h*_HW_ for each locus and population** | | | | | | | | | | |
| --- | --- | --- | --- | --- | --- | --- | --- | --- | --- | --- | --- | --- | --- | --- | --- | --- | --- |
| **Label** | **Population** | ***H_HW_*** | ***P*_FSTAT_** | ***P*_GENEPOP_** | **Global *f*_IS_** |  | **C118** | **C103** | **D115** | **B107** | **C113** | **C9** | **B135** | **A5** | **Pb20** | **Pb55** | **B4** |
| **L1** | Lambre | 0.618 | 0.433 | 0.237 | 0.012 |  | 0.027  6  0.807 | 0.649  2  0.198 | 0.053  4  0.677 | -0.067  3  0.537 | 0.107  4  0.717 | 0.044  6  0.820 | -0.135  4  0.759 | -0.083  2  0.198 | -0.035  3  0.622 | -0.151  5  0.749 | 0.097  6  0.709 |
| **L2** | Mandeo | 0.686 | 0.490 | 0.115 | -0.001 |  | -0.067  4  0.637 | -0.179  7  0.729 | -0.073  8  0.867 | 0.275  5  0.294 | 0.087  5  0.664 | -0.028  5  0.626 | 0.004  5  0.753 | 0.143  5  0.646 | 0.044  8  0.747 | 0.039  6  0.743 | -0.060  9  0.844 |
| **L3** | Capelada | 0.737 | 0.034 | 0.284 | 0.095 |  | 0.200  3  0.450 | 0.162  6  0.861 | -0.064  8  0.857 | 0.070  6  0.779 | 0.030  4  0.749 | -0.136  5  0.805 | 0.146  5  0.740 | 0.605  4  0.671 | 0.037  7  0.848 | 0.216  6  0.688 | -0.119  4  0.654 |
| **L4** | Eume | 0.761 | 0.008 | 0.059 | 0.082 |  | 0.042  5  0.646 | 0.029  7  0.784 | 0.259  11  0.894 | 0.094  7  0.734 | 0.177  5  0.691 | -0.026  5  0.790 | 0.140  8  0.828 | 0.441  9  0.651 | -0.010  8  0.801 | -0.210  6  0.791 | -0.003  7  0.760 |
| **L5** | Sobrado | 0.547 | 0.050 | 0.001 | 0.052 |  | -0.121  4  0.504 | -0.179  3  0.353 | -0.197  6  0.736 | 0.181  2  0.178 | 0.372  5  0.650 | -0.127  3  0.585 | 0.031  3  0.629 | 0.453  4  0.353 | -0.016  5  0.576 | 0.163  4  0.698 | 0.128  5  0.754 |
| **L6A** | Queixa | 0.605 | 0.028 | 0.044 | 0.130 |  | -0.273  3  0.506 | 0.304  3  0.515 | -0.098  5  0.749 | 0.057  3  0.481 | 0.456  4  0.654 | -0.015  3  0.628 | 0.077  3  0.589 | **0.852**  5  0.712 | 0.130  4  0.519 | -0.129  3  0.567 | -0.111  5  0.740 |
| **L7** | Xistral | 0.743 | 0.022 | 0.022 | 0.061 |  | 0.164  5  0.496 | 0.041  7  0.781 | 0.213  7  0.843 | 0.032  6  0.774 | 0.037  5  0.735 | -0.140  8  0.807 | 0.133  8  0.863 | 0.274  5  0.476 | 0.210  9  0.840 | -0.381  5  0.669 | 0.111  12  0.888 |
| **L8A** | Ancares | 0.847 | 0.011 | 0.378 | 0.072 |  | -0.088  8  0.819 | 0.248  7  0.806 | 0.130  8  0.763 | 0.006  9  0.838 | 0.333  8  0.825 | -0.028  8  0.865 | 0.171  9  0.867 | -0.047  12  0.903 | 0.025  9  0.854 | 0.061  7  0.827 | 0.003  16  0.948 |
| **L9A** | Courel | 0.735 | **0.0003** | **0.000** | **0.162** |  | 0.152  7  0.717 | 0.012  7  0.787 | -0.019  8  0.873 | 0.114  8  0.813 | **0.811**  5  0.645 | 0.166  5  0.795 | 0.227  6  0.786 | -0.028  10  0.865 | 0.383  5  0.754 | -0.097  2  0.203 | 0.024  10  0.843 |
| **L9B** | Courel | 0.756 | 0.0006 | 0.001 | 0.152 |  | -0.044  5  0.720 | -0.092  6  0.746 | 0.032  6  0.839 | -0.003  6  0.810 | 0.347  6  0.706 | 0.221  7  0.796 | 0.058  6  0.794 | 0.263  7  0.841 | **0.617**  7  0.800 | 0.096  4  0.413 | 0.124  12  0.853 |
| **L10** | Pindo | 0.637 | 0.671 | 0.853 | -0.012 |  | -0.071  3  0.652 | 0.087  5  0.738 | 0.033  11  0.841 | -0.202  2  0.427 | 0.006  6  0.796 | -0.032  7  0.767 | -0.088  2  0.385 | 0.054  5  0.737 | 0.120  3  0.448 | -0.008  3  0.554 | -0.118  14  0.667 |
| **L11** | Estrela | 0.733 | 0.448 | 0.959 | 0.008 |  | -0.045  6  0.757 | -0.159  7  0.730 | 0.019  12  0.912 | 0.230  8  0.815 | -0.080  5  0.733 | 0.006  5  0.741 | -0.069  8  0.789 | 0.105  8  0.879 | 0.053  10  0.832 | NA  1  0.000 | -0.022  9  0.876 |
| **L13** | Leitariegos | 0.784 | 0.525 | **0.000** | 0.067 |  | 0.003  5  0.705 | -0.055  5  0.667 | 0.054  10  0.828 | -0.032  8  0.838 | 0.122  7  0.799 | -0.071  10  0.884 | 0.044  7  0.735 | 0.168  10  0.713 | **0.540**  10  0.817 | -0.115  4  0.728 | 0.055  14  0.914 |
| **L15** | Vegarada | 0.655 | 0.003 | 0.350 | 0.000 |  | 0.125  5  0.607 | 0.049  6  0.770 | 0.056  7  0.846 | 0.347  5  0.706 | -0.173  5  0.457 | -0.167  6  0.690 | -0.124  4  0.715 | 0.006  4  0.402 | 0.038  4  0.692 | -0.359  2  0.497 | 0.023  7  0.818 |
|  |  |  |  |  |  |  |  |  |  |  |  |  |  |  |  |  |  |

Abbreviations: *f_IS_* inbreeding coefficient; *n*_a_  number of alleles; *h*_HW_, *H*_HW_expected frequency of heterozygotes at HWE per locus, and average across loci, respectively; *P*_FSTAT_, P_GENEPOP_P values after the tests on *f*_IS_ and HWE, carried out with Fstat and Genepop , respectively (see Material and Methods for further details). Significant results at the 5% level after Bonferroni correction (adjusted nominal level = 0.00032) are shown in bold.
